# Synergistic Effect of Waste Glass Powder and Metakaolin on the Microstructure and Mechanical Performance of Cement-Based Pastes and Mortars

**DOI:** 10.3390/ma19061140

**Published:** 2026-03-15

**Authors:** Magnolia Soto-Félix, Fatima J. Espitia-Vázquez, Miguel A. Avila-Rubio, Francisco J. Baldenebro-López, Caleb Carreño-Gallardo, José M. Herrera-Ramírez

**Affiliations:** 1 Facultad de Ingenieria Culiacan, Universidad Autonoma de Sinaloa, Culiacan 80040, Mexico; fatima.espitia.fim@uas.edu.mx; 2 Facultad de Ingenieria Mochis, Universidad Autonoma de Sinaloa, Los Mochis 81223, Mexico; miguel.avila@uas.edu.mx (M.A.A.-R.); francisco.baldenebro@uas.edu.mx (F.J.B.-L.); 3 Centro de Investigación en Materiales Avanzados, S.C. (CIMAV), Av. Miguel de Cervantes #120, Complejo Industrial Chihuahua, Chihuahua 31136, Mexico; caleb.carreno@cimav.edu.mx

**Keywords:** waste glass powder, metakaolin, mechanical properties, microstructure, pore structure refinement, circular economy

## Abstract

**Highlights:**

- MK and WGP act synergistically, improving strength and pore structure over time.
- Gas adsorption reveals reduced accessible pore volume in binary and ternary pastes.
- WGP–MK mortars exhibit lower water absorption and porosity at later curing ages.
- Ternary mortar porosity and absorption decrease significantly from 28 to 60 days.
- Combined WGP–MK systems enable clinker replacement for a circular economy.

**Abstract:**

The incorporation of supplementary cementitious materials (SCMs) is a key strategy for enhancing the performance and sustainability of cement-based systems. This research examines the mechanical behavior, microstructural evolution, and durability-related properties of cementitious materials incorporating waste glass powder (WGP) and metakaolin (MK) as partial replacements of Portland cement. Cement pastes were evaluated for compressive strength at 7 and 28 days, while microstructural analysis at 28 days employed gas adsorption and scanning electron microscopy (SEM). Based on the compressive strength performance of the cement pastes, ternary WGP–MK mortars were assessed for consistency, flexural and compressive strength, water absorption, and porosity at 28 and 60 days. Results indicate that MK accelerates early-age strength, whereas WGP enhances long-term performance and pore structure refinement. Binary and ternary systems exhibited reduced accessible pore volume, enhanced microstructural homogeneity, and lower water absorption with curing time. The findings demonstrate that WGP-MK blends support clinker reduction without compromising performance, advancing circular economy goals in construction.

## 1. Introduction

The cement industry plays a central role in civil infrastructure development, although it remains a primary contributor to anthropogenic CO_2_ emissions [1,2,3]. To mitigate this environmental impact, research has focused on replacing Portland cement with supplementary cementitious materials (SCMs) to enhance resource efficiency, mechanical performance, and long-term durability [4,5,6,7]. Within the framework of the circular economy, the construction sector has increasingly prioritized the valorization of industrial by-products—such as fly ash, coal bottom ash, palm oil fuel ash, sugarcane bagasse ash, and ground granulated blast furnace slag—as viable SCMs [8,9].

Among these materials, waste glass powder (WGP), obtained from discarded glass products, has emerged as a promising candidate due to the substantial global quantities of glass waste and its high amorphous silica content. When finely ground, WGP exhibits pozzolanic reactivity, making it suitable for cement-based systems [10,11].

The extensive literature suggests that WGP contributes to late-age strength development and matrix densification through the formation of additional calcium silicate hydrate (C–S–H) phases [12,13]. However, its pozzolanic reactivity is highly sensitive to particle size, curing conditions, and replacement level. While some research highlights densification benefits, others suggest that WGP’s early-age contribution is limited to a physical filler effect rather than true pozzolanic activity [14,15]. From a sustainability perspective, the incorporation of WGP represents a viable strategy for its integration into cement-based materials [16].

In parallel, metakaolin (MK) is widely recognized as a highly reactive aluminosilicate supplementary cementitious material produced through the calcination of kaolinitic clays [17,18]. Its incorporation into cementitious systems typically accelerates hydration, increases calcium hydroxide consumption, and refines the pore structure, resulting in improved early-age mechanical and durability properties [19,20]. Unlike WGP, MK is a manufactured performance enhancer that can complement slower-reacting materials [21,22].

Recent findings indicate that combining WGP and MK may yield a synergistic effect: MK compensates for the initial slow reactivity of WGP, while WGP ensures continued microstructural densification and strength gain over time [10,23]. Experimental investigations on ternary WGP–MK systems have explored a wide range of replacement proportions and performance responses. Reported MK contents typically vary between 5 and 15% as cement replacement, while WGP replacement levels range from 5 to 20%, depending on particle fineness and intended performance criteria [24,25,26]. Despite these efforts, research has primarily focused on macroscopic strength, often overlooking the synchronized evolution of pore-size distribution and quantitative hydration kinetics over extended curing ages.

In binary systems, MK has demonstrated early-age strength enhancement due to rapid portlandite consumption and the formation of C–A–S–H gels, whereas WGP-based binders tend to exhibit delayed pozzolanic activity and strength development at later curing ages [27]. When combined in ternary systems, this dual-pathway activation promotes a complementary mechanism [28]: the alumina-rich contribution of MK promotes early nucleation and hydration acceleration, while the gradual silica dissolution from WGP sustains secondary C–S–H formation at later ages [29]. However, a consensus on the optimal synergy to mitigate the dilution effect while enhancing long-term durability remains an open area of discussion in the context of high-performance cementitious composites.

Despite these advancements in WGP and MK as SCMs, significant knowledge gaps remain. There is a lack of integrated studies that simultaneously bridge the gap between hydration mechanisms at the paste level and macroscopic performance in mortars. Furthermore, durability-related properties—such as porosity, water absorption and density—are frequently analyzed in isolation, without establishing a direct correlation with microstructural evolution across different curing stages.

To address these gaps, this study provides a comprehensive evaluation of cementitious materials incorporating both recycled WGP and MK. The research systematically assesses the mechanical performance and microstructural evolution of cement pastes through compressive strength testing, X-ray diffraction (XRD), thermogravimetric and differential scanning calorimetry analyses (TGA–DSC), gas adsorption analysis, and scanning electron microscopy (SEM). Based on these findings, ternary mortars were evaluated in terms of consistency, flexural and compressive strength, density, water absorption, and porosity at different curing ages. By framing the valorization of glass waste within a circular economy perspective and identifying the complementary role of metakaolin, this work advances the understanding of blended SCM systems for sustainable construction.

## 2. Materials and Methods

### 2.1. Materials

Portland cement (PC), WGP, and MK served as the primary precursors, with distilled water used for mixture preparations. Commercial PC (CEMEX, Hermosillo, Mexico) and MK (GCC, Chihuahua, Mexico) were used as received, while WGP was sourced from post-consumer glass waste and processed to achieve the required fineness. The raw WGP, with an initial maximum particle size of 75 µm, was subjected to high-energy milling and was subsequently sieved through a No. 325 (45 µm) mesh. For mortar specimens, graded sand conforming to ASTM C778 [30] was used as fine aggregate.

The chemical composition of the precursor materials, determined by energy-dispersive X-ray fluorescence (XRF) using an Epsilon 3XLE spectrometer (Malvern Panalytical, Almelo, The Netherlands), is summarized in Table 1. WGP is characterized by a high SiO_2_ content (71.0%), typical of soda–lime glass. MK exhibits elevated Al_2_O_3_ (26.2%) and SiO_2_ (54.8%) contents, consistent with the controlled calcination of kaolinitic clays. Particle size distributions were measured by laser granulometry using a CILAS 1190L analyzer (CILAS, Orléans, France). The characteristic particle diameters d10, d50, and d90 are summarized in Table 2. The median particle size (d50) for both PC and WGP was approximately 10 µm, whereas MK exhibited a slightly higher value of 12.1 µm. Differences in particle size distribution are expected to influence particle packing, hydration kinetics, and microstructural development in the blended cementitious systems.

**Figure 1:**
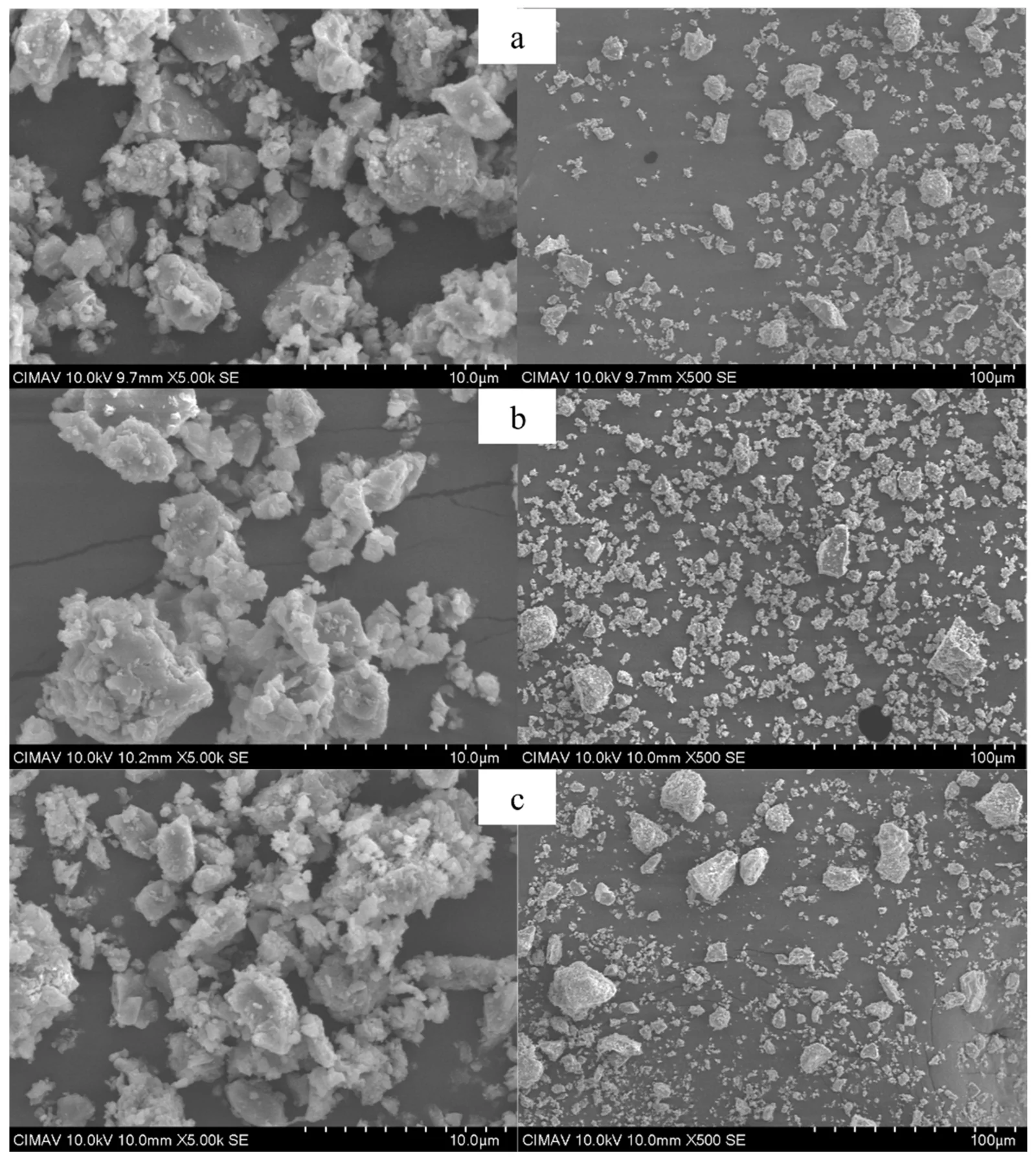
SE-SEM micrographs of precursor materials: (**a**) PC, (**b**) WGP, and (**c**) MK.

The morphological characteristics of the precursor materials were analyzed by scanning electron microscopy (SEM) using a Hitachi SU3500 microscope (Hitachi High-Technologies Corporation, Tokyo, Japan), operated at an accelerating voltage of 10 kV. Representative secondary electron micrographs (SE-SEM) of PC, WGP and MK are presented in Figure 1. Morphological analyses revealed that PC particles exhibit angular to sub-angular morphologies with relatively smoother surfaces, typical of ground clinker phases [31]. WGP particles exhibit angular and irregular morphologies with sharp edges and fractured surfaces, characteristic of mechanically ground glass [25]. In contrast, MK particles display finer morphologies with plate-like and lamellar features, commonly associated with the calcination of kaolinitic clays as reported in previous SEM studies [32].

### 2.2. Methods

Cement pastes were prepared by replacing Portland cement with various levels of WGP and MK, as detailed in Table 3. The mixtures were prepared following the ASTM C305 standard [33] to ensure homogeneity, maintaining a constant water-to-cementitious materials (w/cm) ratio of 0.32. After casting, the specimens were sealed for 24 h to prevent moisture loss. Upon demolding, they were cured in a saturated calcium hydroxide (CH) solution at room temperature to prevent leaching and ensure optimal hydration until the testing ages.

Compressive strength was determined at 7 and 28 days of curing in accordance with ASTM C109/C109M [34] using an INSTRON 600DX universal testing machine (INSTRON, Norwood, MA, USA). Prior to testing, the specimens were removed from the curing solution and surface dried. The reported values constitute the mean of three independent replicates.

The experimental program was strategically structured into two distinct phases: (i) a preliminary screening phase and (ii) an in-depth characterization phase. The first stage involved a systematic evaluation of five paste formulations (binary and ternary) under identical curing conditions to identify the synergistic threshold of the WGP–MK system. This screening approach was designed to isolate the binder configuration that maximized clinker replacement without compromising mechanical integrity.

Based on the compressive strength hierarchy observed in the pastes, the 10WGP5MK formulation was identified as the optimal candidate, demonstrating the most effective balance between pozzolanic reactivity and waste incorporation. Consequently, this specific ternary system was selected for the second phase, which comprised comprehensive physicochemical and microstructural analyses, as well as the evaluation of performance at the mortar level. This sequential methodology ensures that the detailed microstructural evolution and durability indicators (water absorption and porosity) are representative of the most promising binder for sustainable construction applications.

For the selected mixture, representative fragments were extracted from the inner core of fractured specimens in order to minimize the influence of surface carbonation and external contamination. To preserve the hydration state at the selected curing age, the samples were immersed in isopropyl alcohol for 24 h to arrest further hydration, followed by drying at 55 °C for an additional 24 h prior to microstructural characterization.

XRD analysis was carried out using a Bruker D8 Advance diffractometer (Bruker Corporation, Billerica, MA, USA) equipped with Cu Kα radiation (λ = 1.5408 Å). This technique was employed to identify anhydrous clinker phases and hydration products, as well as to evaluate the influence of WGP and MK on phase assemblage and microstructural evolution. Phase identification and indexing were performed using X’Pert HighScore Plus software (v. 3.0, Malvern Panalytical, Almelo, The Netherlands).

Thermogravimetric and differential scanning calorimetry analyses (TGA/DSC) were conducted using an SDT Q600 thermal analyzer (TA Instruments, New Castle, DE, USA) under an inert argon atmosphere. Samples were heated at a constant rate of 10 °C/min from room temperature up to 900 °C. The weight-loss events and corresponding thermal effects associated with the decomposition of hydration products were analyzed to assess the influence of WGP and MK on cement hydration and phase evolution. To provide a precise quantitative assessment of the hydration kinetics and the pozzolanic activity of the WGP–MK system, the CH content was determined from the thermogravimetric (TG) data. The CH percentage, *CH* (%), was calculated based on the mass loss (*WL_CH_*) occurring during the dehydroxylation of portlandite, typically observed between 400 °C and 500 °C, using the stoichiometric relationship of Equation (1) [26].

(1) $$CH\left(\%\right)={WL}_{CH}\left(\%\right)\times \frac{{MW}_{Ca{\left(OH\right)}_{2}}}{{MW}_{H_{2}O}}$$

Here, *MW_Ca_*_(*OH*)2_ (74.09 g/mol) and *MW_H_*_2*O*_ (18.01 g/mol) are the molecular weights of calcium hydroxide and water, respectively. To ensure a rigorous comparison between the different binder systems and to account for the substitution levels, all CH values were normalized to the anhydrous weight of the cement in the starting mixture. This quantification allows for the isolation of the pozzolanic consumption of CH from the simple dilution effect caused by the incorporation of WGP and MK.

The pore structure of the hardened cement pastes was evaluated by physical gas adsorption using a Quantachrome NovaWin analyzer (Quantachrome Instruments, Boynton Beach, FL, USA). Before testing, samples were degassed at 250 °C for 12 h to remove physically adsorbed moisture. The specific surface area was calculated using the Brunauer–Emmett–Teller (BET) method, while pore-size distribution and total pore volume were obtained through Barrett–Joyner–Halenda (BJH) analysis.

Complementary microstructural characterization was conducted using SEM. Prior to analysis, the samples were gold-coated to ensure adequate electrical conductivity. Examinations were performed at accelerating voltages between 5 and 10 kV in secondary electron mode. Micrographs were acquired at various magnifications to assess the morphology and distribution of hydration products, as well as the interfacial interaction between the cement matrix and the incorporated WGP and MK.

Mortars were prepared to evaluate the performance of the optimized formulation identified during the paste-level screening stage. This phase focused on the 10WGP5MK ternary system (10% WGP and 5% MK), which demonstrated the most favorable balance between mechanical performance and clinker replacement during the preliminary assessment. The mixture proportions were designed in accordance with ASTM C109/C109M [34], using a cement-to-sand ratio of 1:2.75 by mass and a constant water-to-cementitious materials (w/cm) ratio of 0.585. Two specific mortar systems were produced for comparison: a control mixture (100% PC) and the selected ternary mixture (10WGP5MK). To ensure that the incorporation of SCMs did not compromise workability, the consistency of the fresh mortars was evaluated via flow table tests following ASTM C1437 [35]. The results indicated comparable rheological behavior, with flow diameters of approximately 205–206 mm (flow values of 105–106%), confirming that the ternary combination maintains adequate flowability for casting without the need for additional chemical admixtures.

Fresh mortars were cast into steel molds measuring 40 × 40 × 160 mm. After casting, the specimens were covered and stored under laboratory conditions for 24 h, then demolded and cured under the same conditions as the cement pastes. Flexural strength tests were performed on the prismatic specimens at curing ages of 7, 28, and 60 days in accordance with ASTM C348 [36]. Following flexural testing, the two halves obtained from each prism were used for compressive strength determination at the same curing ages in accordance with ASTM C349 [37].

Water absorption and porosity tests were conducted on mortar cubes with dimensions of 50 × 50 × 50 mm at curing ages of 28 and 60 days, following the procedure described in ASTM C642 [38]. Although this standard was originally developed for hardened concrete, its methodology is suitable for application to mortar specimens of comparable geometry, such as 50 mm cubes, and has been widely adopted for evaluating absorption and permeable porosity in cement-based materials. These tests were performed to evaluate the influence of WGP and MK incorporation on the physicomechanical and durability-related properties of the mortars.

## 3. Results and Discussion

This section provides a detailed discussion of the experimental results obtained from the physical, chemical, structural, and microstructural characterization of the cementitious systems. The following subsections discuss the impact of individual and combined additions of WGP and MK on the mechanical performance, phase evolution, and pore structure refinement of both pastes and mortars. By integrating macroscopic tests with advanced instrumental techniques, the synergistic effect between these materials is elucidated, highlighting their potential for developing more sustainable and durable construction materials.

### 3.1. Compressive Strength of Pastes

**Figure 2:**
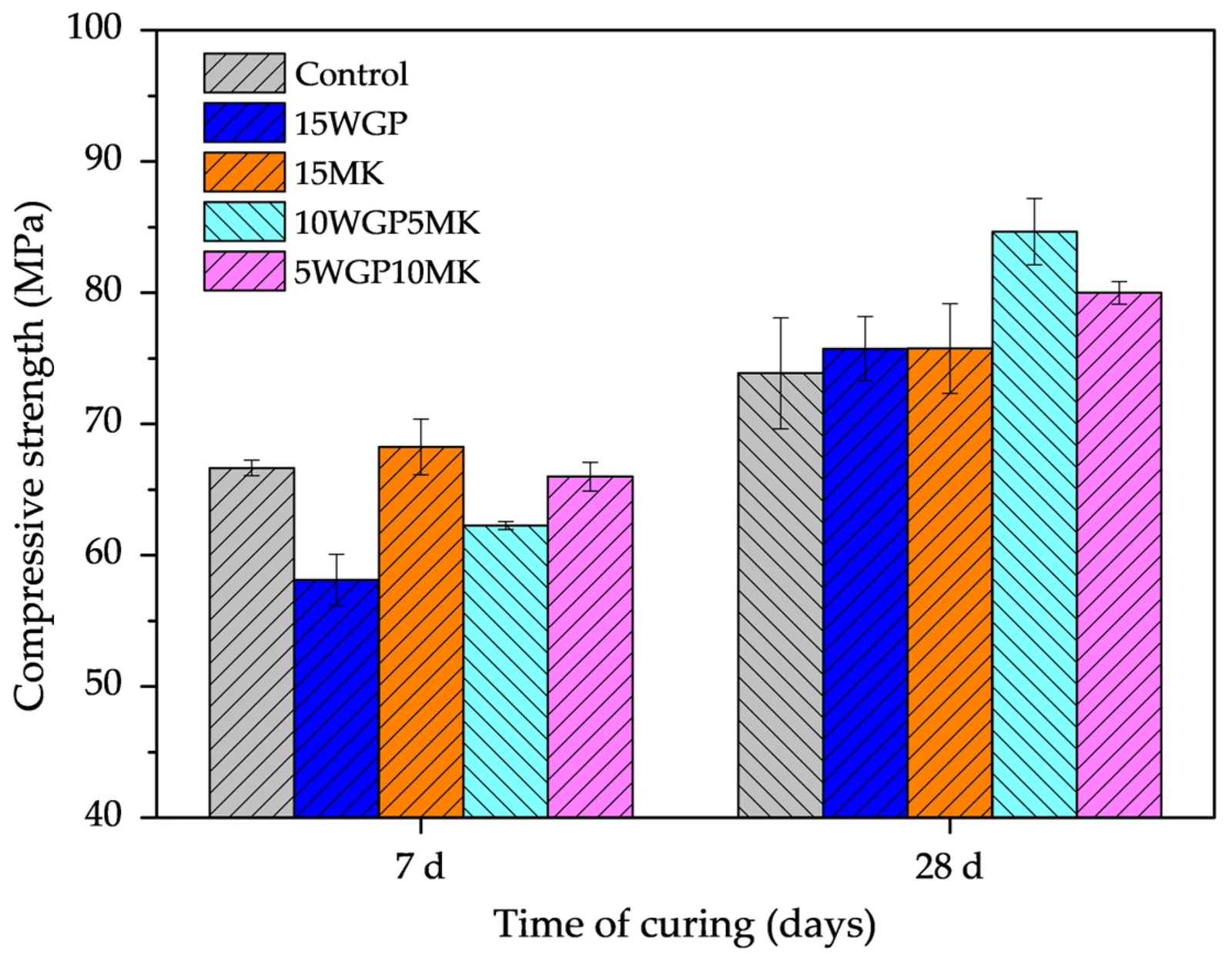
Compressive strength of studied pastes at 7 and 28 curing days.

The compressive strength results (Figure 2) demonstrate contrasting influences of MK and WGP on strength development at early and later curing ages. At 7 days, the paste containing 15 wt.% MK (15MK) exhibits the highest compressive strength (68.26 MPa) among the modified mixtures, surpassing that of the control paste (66.64 MPa). This early-age enhancement is attributed to the high pozzolanic reactivity of MK, which is associated with its elevated amorphous aluminosilicate content. The accelerated dissolution of reactive Al_2_O_3_ and SiO_2_ phases accelerates portlandite consumption and promotes the formation of C–(A)–S–H gels with reduced Ca/Si ratios, leading to early microstructural densification and improved mechanical performance. In contrast, the paste containing 15 wt.% WGP (15WGP) shows the lowest compressive strength at 7 days (58.11 MPa), reflecting the delayed pozzolanic activity of glass powder, which requires sustained alkaline conditions and extended curing time to activate silica dissolution.

At 28 days, a marked increase in compressive strength is observed for all WGP-containing systems. The paste with 15 wt.% WGP (15WGP) reaches 75.73 MPa, exceeding the control mixture (73.86 MPa), which confirms the progressive contribution of WGP through the formation of secondary C–S–H phases. Notably, the mixture containing 10 wt.% WGP and 5 wt.% MK (10WGP5MK) exhibits one of the highest compressive strength values at 28 days (79.99 MPa), highlighting the beneficial interaction between MK and WGP.

The comparative evolution of these pastes highlights a synergistic threshold in the ternary system. While 15MK dominates at 7 days due to the rapid formation of C–A–S–H gels and immediate portlandite consumption, the 10WGP5MK formulation provides a more balanced hydration profile over time. This suggests that in MK-rich mixtures (like 15MK and (5GWP10MK), the system may experience a reactive plateau or a faster depletion of available Ca(OH)_2_. In contrast, the 10WGP5MK system benefits from a dual-pathway mechanism [28]: the alumina-rich MK triggers early-age reactivity, while the gradual silica dissolution from WGP sustains secondary C–S–H formation at later stages, effectively refining the matrix and enhancing the 28-day strength.

From a practical and environmental perspective, the selection of 10WGP5MK as the optimal binder is further justified by its superior material efficiency. Replacing a portion of MK (which requires energy-intensive calcination) with WGP (an untreated industrial waste) reduces the embodied carbon footprint without compromising mechanical integrity. These findings align with previous studies [22,25], where ternary MK–WGP combinations outperformed binary counterparts due to the complementary acceleration of hydration products. Consequently, the 10WGP5MK system represents the ideal balance between early-stage reactivity, long-term strength, and sustainability, serving as the technical basis for the subsequent mortar-scale evaluation and microstructural analysis.

### 3.2. Structural and Thermal Characterization

**Figure 3:**
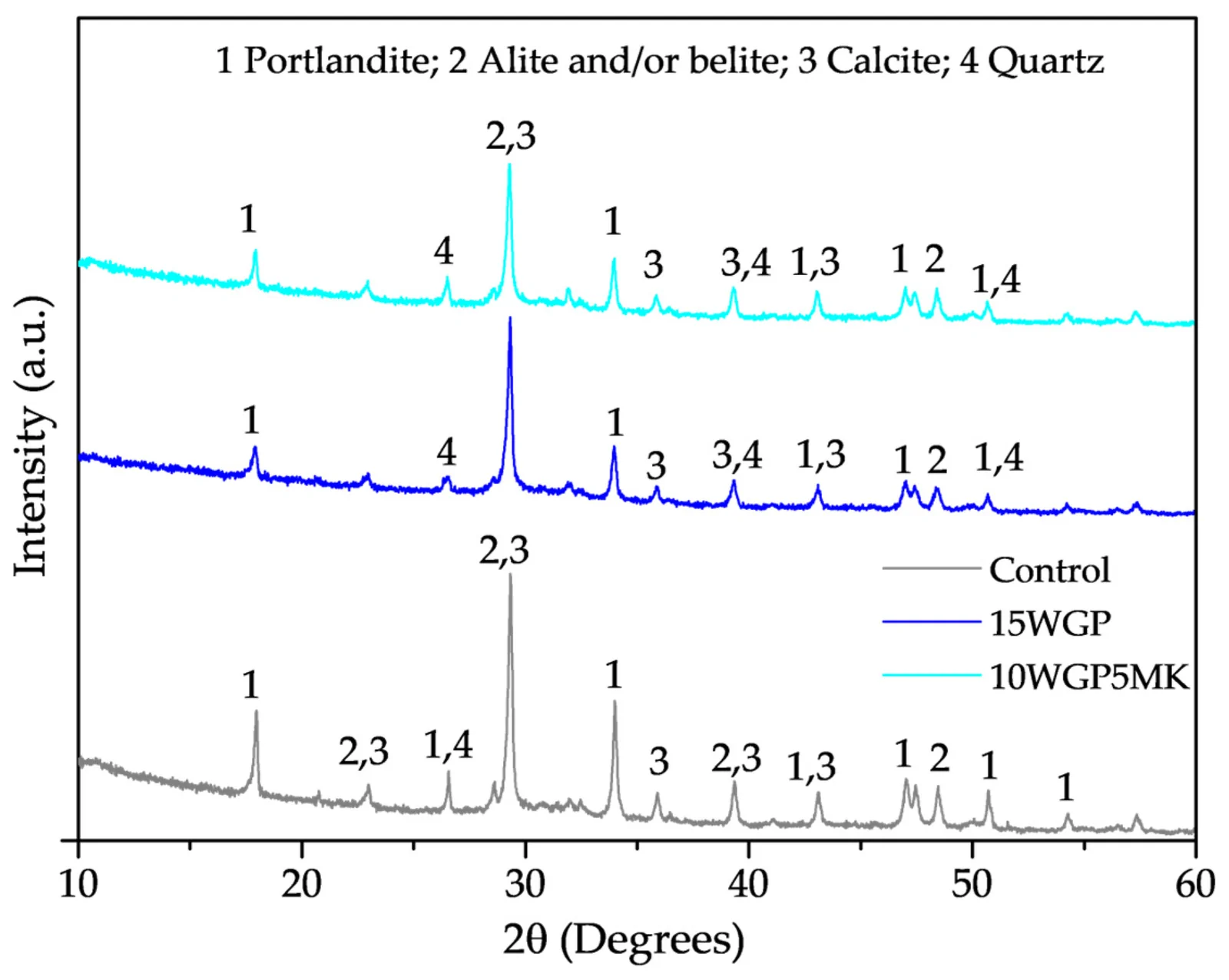
XRD patterns of studied pastes cured at 28 days.

The XRD patterns of the cement pastes cured at 28 days (Figure 3) reveal the typical crystalline phases associated with hydrated Portland cement systems. In the control paste, the main identified phases correspond to portlandite (CH; PDF 01-084-1265), residual alite and/or belite (C_3_S/C_2_S; PDF 01-086-0402 and 00-001-1012), calcite (CaCO_3_; PDF 01-072-1937), and quartz (SiO_2_; PDF 01-081-0065). The main portlandite reflections are clearly observed at 2θ ≈ 18.0°, 34.1°, and 47.1°, indicating normal hydration of clinker phases. Calcite peaks, mainly located around 29.4°, are associated with limestone additions and partial carbonation of hydration products. Residual alite and/or belite reflections, as well as quartz peaks, are observed in all three systems (control, 15WGP, and 10WGP5MK pastes), indicating the presence of unreacted clinker phases and residual crystalline silica.

In the pastes containing WGP, either alone (15WGP) or combined with MK (10WGP5MK), a noticeable reduction in the intensity of portlandite reflections is observed compared to the control paste, particularly at 2θ ≈ 18° and 34°. This trend suggests the progressive consumption of calcium hydroxide associated with pozzolanic reactions involving the reactive silica of the glass powder and, in the case of 10WGP5MK, the additional contribution of reactive aluminosilicates from metakaolin [39]. Calcite and quartz reflections remain present in both systems. In addition, residual alite and/or belite peaks are still detected at 28 curing days [40]; however, their relative intensity is lower than in the control paste, suggesting a more advanced degree of clinker hydration in the blended systems at the analyzed curing age.

**Figure 4:**
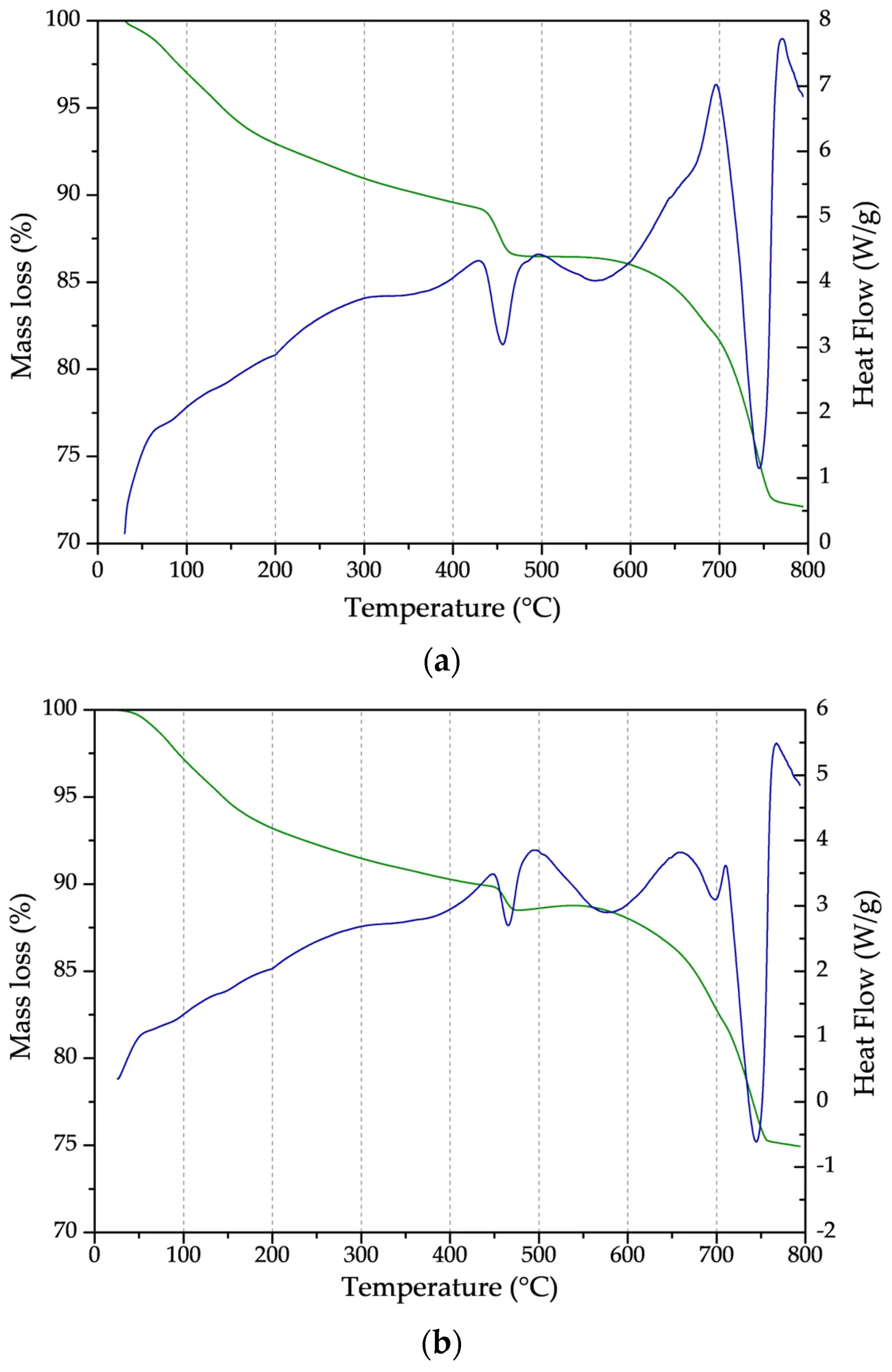

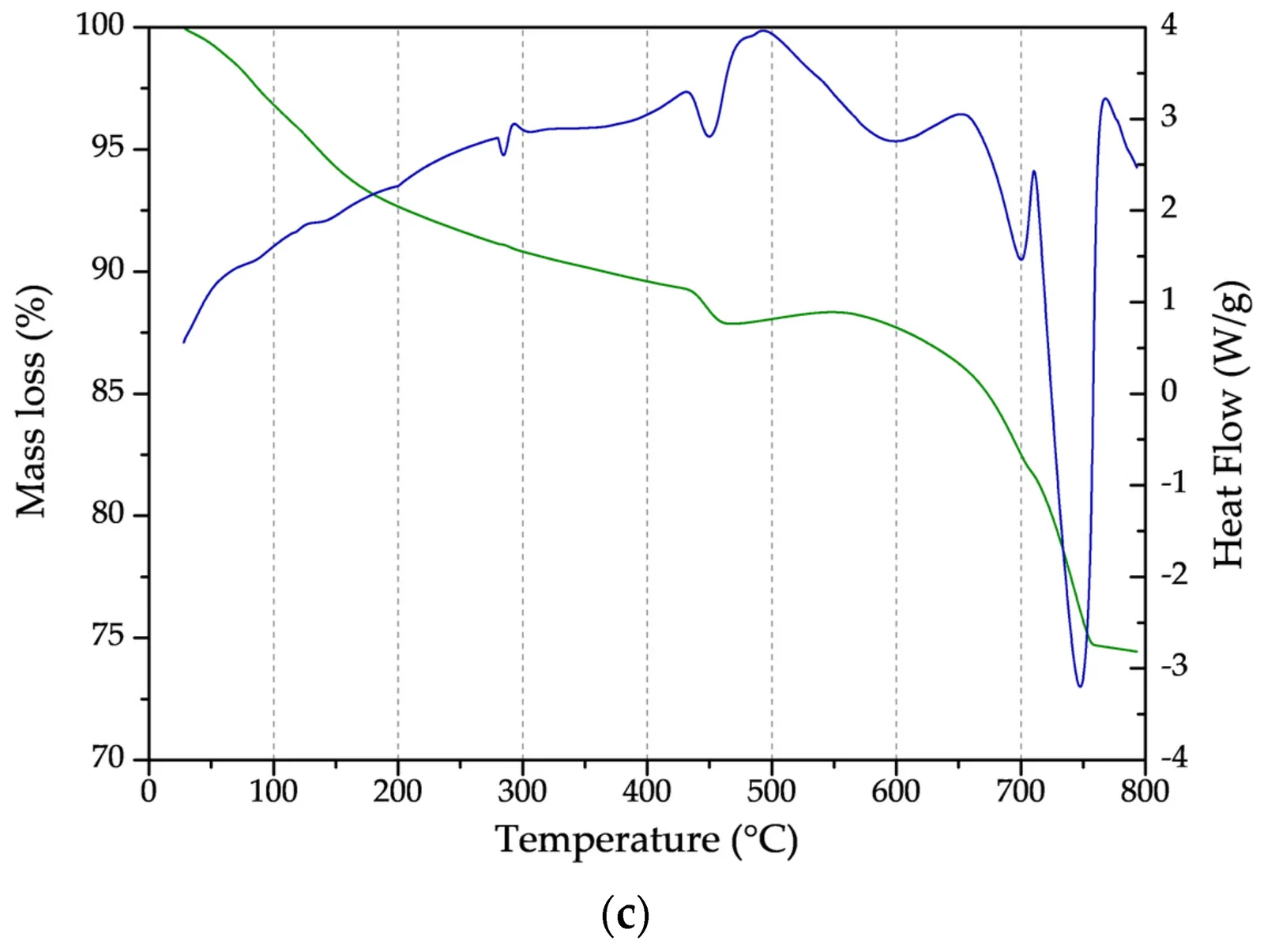
TGA analysis of (**a**) control, (**b**) 15WGP, and (**c**) 10WGP5MK pastes cured at 28 days. The green lines represent TGA data (left y-axis) and the blue lines correspond to the DSC data (right y-axis).

The TGA/DSC curves of the cement pastes cured for 28 days are presented in Figure 4. All mixtures exhibit characteristic weight-loss stages associated with the dehydration and decomposition of hydration products in Portland cement systems.

For the control paste (Figure 4a), three main weight-loss regions can be identified. The first mass loss below approximately 200 °C is attributed to the removal of physically bound water and the dehydration of C–S–H gel and ettringite. The second weight-loss event, occurring between 400 and 500 °C, corresponds to the dehydroxylation of portlandite, which is accompanied by a distinct endothermic peak in the DSC curve. The third weight-loss stage, observed between 600 and 750 °C, is associated with the decarbonation of calcium carbonate, mainly calcite, also reflected by a pronounced endothermic DSC signal.

In the paste containing 15 wt.% WGP (15WGP) (Figure 4b), the overall thermal decomposition profile remains similar to that of the control paste; however, a noticeable reduction in the mass loss associated with portlandite decomposition is observed in the 400–500 °C range, together with a lower intensity of the corresponding DSC endothermic peak, indicating a reduced CH content. The mass loss at low temperatures, associated with bound water, remains significant, suggesting the presence of hydration products such as C–S–H and related secondary C–S–H phases formed through pozzolanic reactions [41].

For the paste incorporating 10 wt.% WGP and 5 wt.% MK (10WGP5MK) (Figure 4c), a further decrease in the mass loss associated with portlandite dehydroxylation is observed, which is also reflected by a weaker DSC endothermic peak in the same temperature range. This reduction is accompanied by a relatively higher mass loss at temperatures below 400 °C, indicating an increased amount of bound water associated with poorly crystalline hydration products, such as C–S–H, secondary C–S–H, and C–A–S–H [42].

Decarbonation was observed at high temperatures across all mixtures. However, the DSC curves of the blended pastes exhibited a more complex profile, characterized by a shoulder preceding the main peak at approximately 700–750 °C. This behavior may be attributed to the modified hydration assemblage in the SCM-bearing systems, which facilitates the formation of secondary carbonate phases before the primary calcite decarbonation event [43].

As summarized in Table 4, the blended pastes show a marginal increase in mass loss within the 110–400 °C temperature range compared to the control mixture. This interval is primarily associated with the progressive dehydration of calcium silicate hydrate (C–S–H) and other aluminosilicate hydration phases, such as C-A-S-H. The increased mass loss in this region for the modified pastes suggests a higher content of chemically bound water, which correlates with the formation of additional hydration products derived from the pozzolanic reactions of WGP and MK.

TGA results revealed a significant reduction in CH content within the blended systems, evidencing high pozzolanic activity. While the control mixture exhibited the highest CH content (12.74 wt.%), the binary system (15 wt.% WGP) showed a decrease to 6.99 wt.%. Notably, the ternary 10WGP5MK formulation reached the lowest CH content (6.17 wt.%), representing reductions of approximately 45% and 52% relative to the control paste. This marked decrease in CH—normalized to the anhydrous cement weight—confirms the synergistic efficiency of the WGP-MK system in consuming portlandite to refine the cementitious matrix.

Overall, the combined XRD and TGA/DSC analyses demonstrate that the partial replacement of Portland cement with WGP and MK reduces portlandite content and promotes the formation of additional poorly crystalline hydration products [44]. These changes in phase assemblage and thermal behavior are consistent with enhanced microstructural development and the improved mechanical performance observed in the blended cement pastes, particularly the higher compressive strength attained by 15WGP and, more markedly, by 10WGP5MK at 28 days, confirming the beneficial role of additional C-(A)-S-H formation in mechanical behavior.

### 3.3. Porosity and Microstructural Analysis of Pastes

Physical gas adsorption results (Table 5) reveal that the incorporation of WGP and MK significantly modifies the pore structure by 28 days. The control paste exhibited the highest specific surface area (18.42 m^2^/g) and total pore volume (0.0747 cm^3^/g), reflecting a higher fraction of accessible pores within the reference cementitious matrix.

According to the IUPAC classification [45], pores are categorized as micropores (<2 nm), mesopores (2–50 nm), and macropores (>50 nm). The average pore sizes obtained from gas adsorption analysis range between approximately 7.9 and 8.8 nm, indicating that the pore systems of all studied pastes are predominantly composed of mesopores. In hydrated cementitious systems, pores within this size range are generally associated with gel pores and small capillary pores within the C–S–H structure, which exert a critical influence on transport properties and mechanical performance.

The incorporation of 15 wt.% WGP leads to a substantial reduction in both parameters, resulting in a specific surface area of 12.39 m^2^/g and a total pore volume of 0.0489 cm^3^/g. This reduction indicates a clear densification of the pore network at 28 days, which can be attributed to the progressive pozzolanic reaction of the amorphous silica in the glass powder. The secondary C–S–H produced during this reaction partially fills the larger capillary pores, effectively refining the internal pore structure of the cement matrix.

The ternary system containing 10 wt.% WGP and 5 wt.% MK exhibits intermediate values for specific surface area (13.64 m^2^/g) and total pore volume (0.0599 cm^3^/g), suggesting a combined microstructural effect. While the total accessible pore volume remains lower than that of the control paste, the average pore size of the 10WGP5MK mixture (8.78 nm) is slightly higher than that of the control (8.11 nm). This behavior suggests that the modification of the pore system is not a simple refinement but a complex redistribution within the mesoporous range, involving changes in the relative proportion of gel pores and small capillary pores [46].

The incorporation of MK contributes to the formation of C–A–S–H-type phases due to its high alumina content, which accelerates early microstructural densification. Conversely, WGP provides reactive silica that promotes the formation of additional C–S–H during later hydration stages. The interaction of these two mechanisms results in a progressive evolution of the pore structure and thereby reducing the total accessible porosity while modifying the distribution of mesopores within the hydrated matrix.

Similar pore refinement trends have been reported in binary PC–MK systems, where metakaolin incorporation significantly reduces capillary pore volume and enhances the formation of C–A–S–H gels, resulting in a denser pore network and improved microstructural compactness [19]. Likewise, cementitious materials containing waste glass powder as partial cement replacement have shown progressive modification of the pore structure and a reduction in accessible porosity due to secondary C–S–H formation [47]. In this context, the behavior observed in the present ternary PC–WGP–MK system can be interpreted as the integration of both mechanisms, combining early alumina-driven densification with delayed silica-driven pore refinement.

The modification of the pore structure and its redistribution within the mesopore range contribute to the overall densification of the cement matrix. This evolution of the pore network reduces the connectivity of capillary pores, thereby limiting transport pathways for fluids and aggressive ions. Such microstructural changes are consistent with the enhanced compressive strength observed in the blended systems and are commonly associated with improved durability in cementitious materials incorporating reactive supplementary cementitious materials.

**Figure 5:**
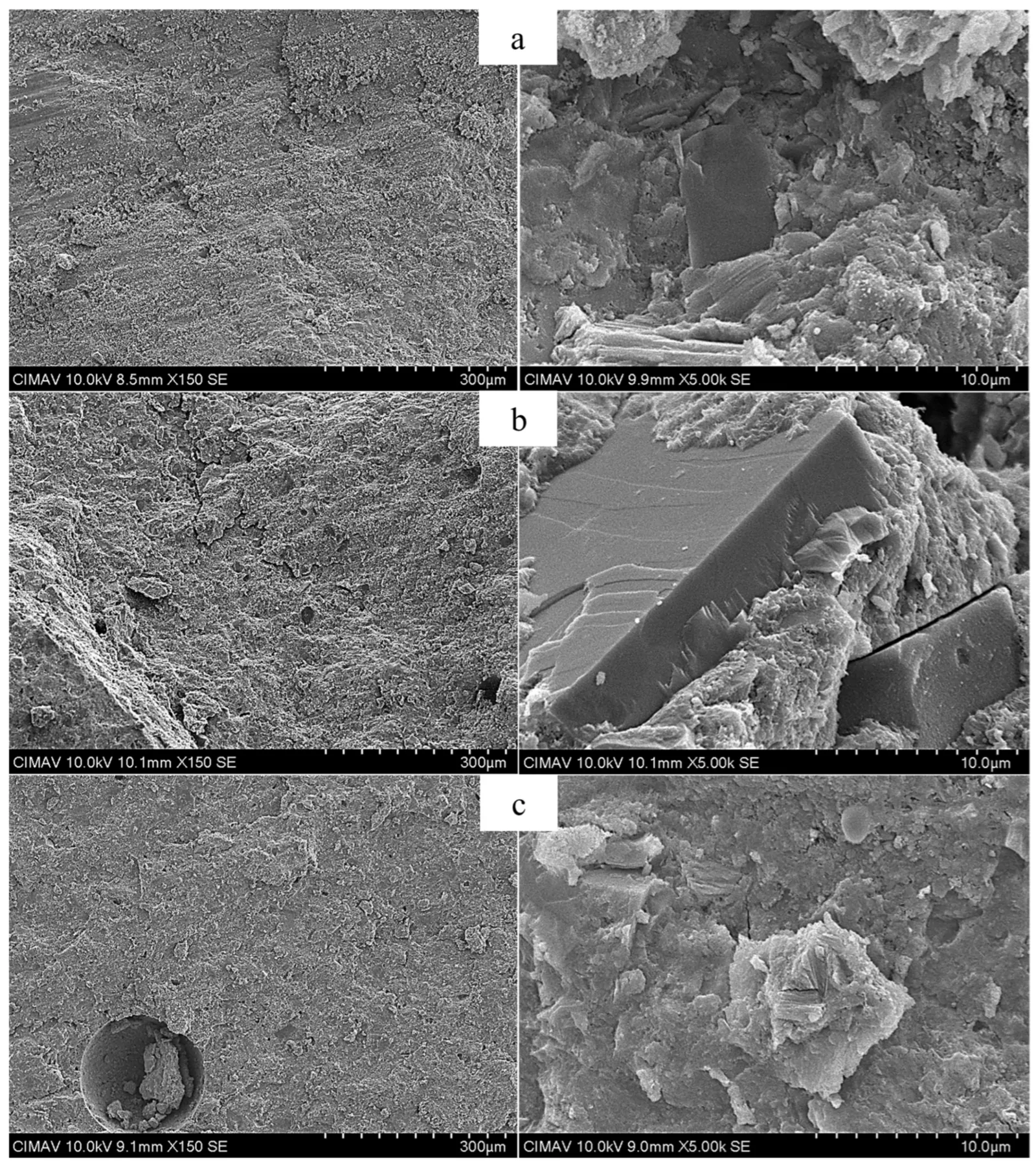
SE-SEM micrographs of pastes: (**a**) control, (**b**) 15WGP, and (**c**) 10WGP5MK.

SEM observations (Figure 5) provide visual evidence. The control paste (Figure 5a) exhibits a heterogeneous surface with microcracks. The paste incorporating WGP (Figure 5b) exhibits a microstructure comparable to that of the control paste, with the presence of angular glass particles embedded in the cementitious matrix. Notably, the ternary paste incorporating both WGP and MK (Figure 5c) displays the most homogeneous and compact microstructure with a continuous matrix and fewer voids. This enhanced homogeneity, despite the intermediate total pore volume measured by gas adsorption, suggests that the ternary cement–WGP–MK system promotes effective spatial distribution and packing of hydration products at the microscale, rather than solely reducing the total accessible porosity.

The combined interpretation of porosity measurements and SEM observations indicates that while the binary WGP system is more effective in reducing total accessible pore volume, the ternary WGP–MK system leads to a more homogeneous and compact microstructural arrangement. This distinction is consistent with the mechanical performance of the blended systems and reflects the complementary roles of WGP and MK in modifying the cement matrix.

### 3.4. Fresh, Mechanical and Durability-Related Properties of Mortars

The results of the fresh and hardened state tests performed on the mortars prepared with the selected ternary binder (WGP–MK) and the control mixture are discussed in this section. The ternary mortar was selected based on the favorable performance observed at the paste level, particularly in terms of mechanical strength development and microstructural characteristics.

The incorporation of WGP and MK resulted in a slight reduction in mortar consistency compared to the control mixture. The measured flow diameter decreased by approximately 1%; however, the obtained value remained within the permissible range established by the standard (110 ± 5%). This indicates that the combined use of WGP and MK does not adversely affect the fresh behavior of the mortars nor significantly increase water demand. The minor reduction in flowability is mainly associated with the replacement level and particle characteristics of WGP and MK, while maintaining adequate workability and mix stability. Similar trends have been reported in previous studies involving blended mortars with WGP additions [48].

**Figure 6:**
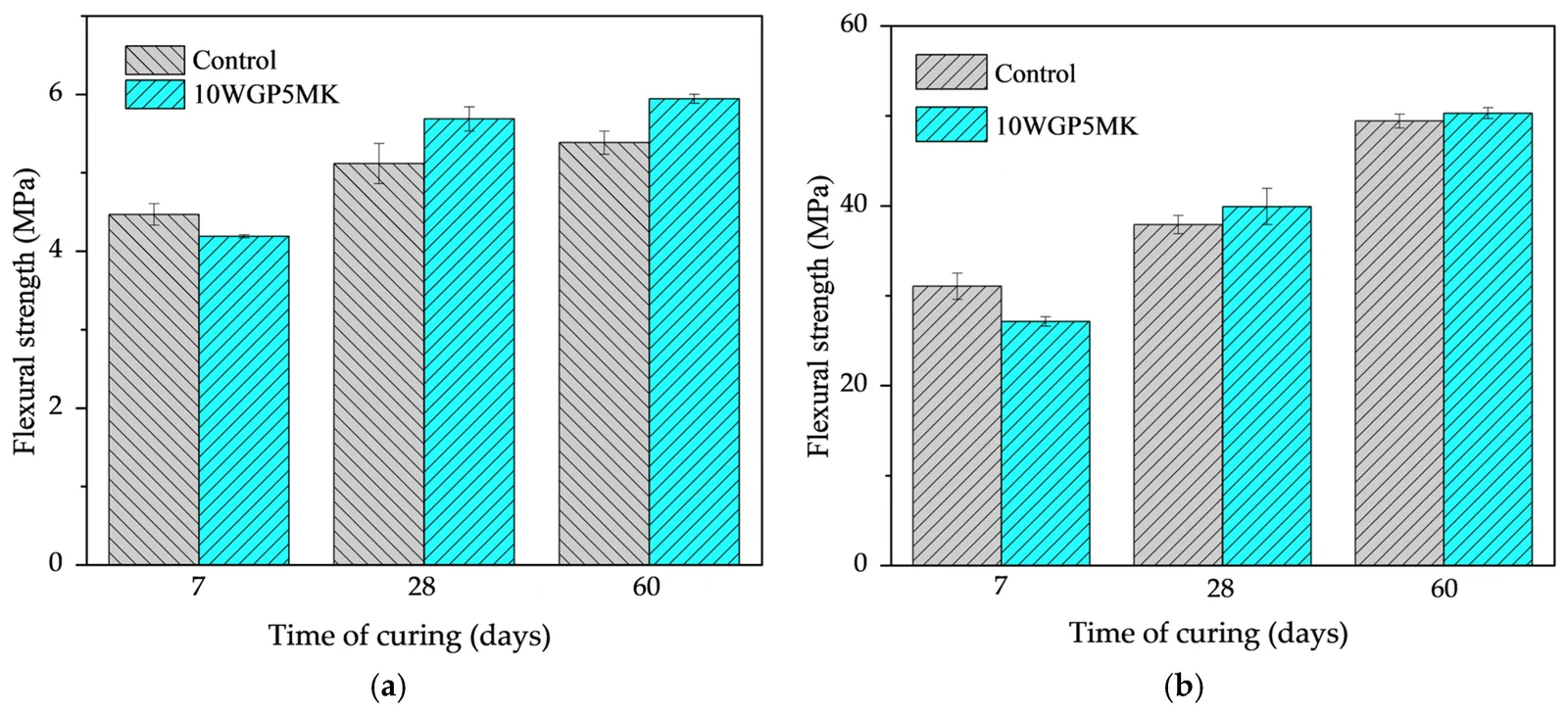
Mechanical properties of mortars: (**a**) flexural and (**b**) compressive strength.

The flexural and compressive strength results of the mortars at 7, 28, and 60 curing days are presented in Figure 6. At an early curing age (7 days), the control mixture exhibits slightly higher flexural and compressive strength than the mortar containing WGP and MK. From 28 days onward, the mortar incorporating WGP and MK shows a slight but consistent increase in both flexural and compressive strength compared to the control mixture. This behavior is attributed to the progressive pozzolanic activity of WGP and MK, which leads to the formation of additional C–(A)–S–H gels and contributes to continued strength development at later ages. The delayed contribution of WGP, combined with the higher reactivity of MK, promotes a synergistic effect that enhances mechanical performance over time. Similar strength development trends have been reported for mortars incorporating glass powder and metakaolin at w/cm ratios close to 0.5 [49,50].

**Figure 7:**
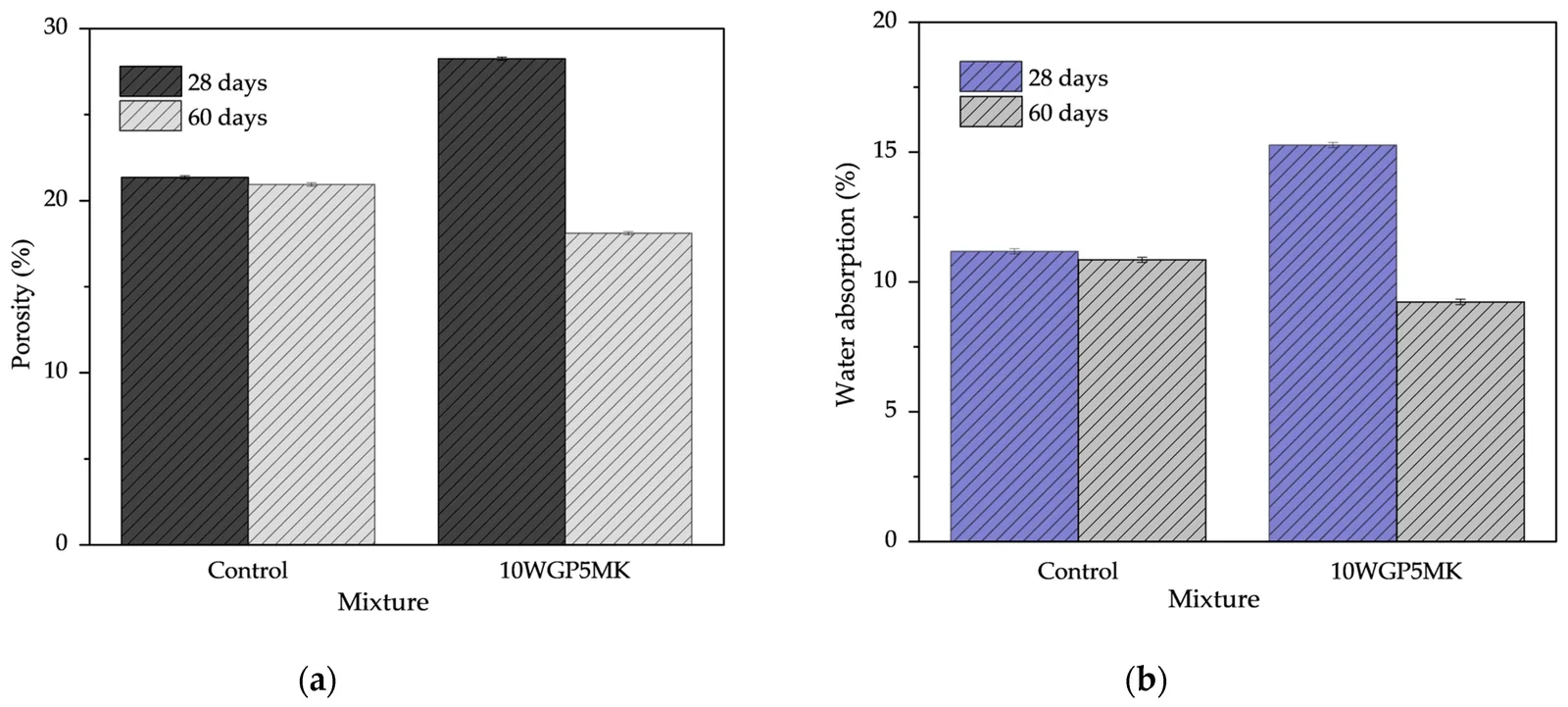
Durability-related properties of mortars: (**a**) porosity and (**b**) water absorption.

Beyond mechanical performance, durability-related properties were evaluated to further assess the influence of WGP and MK on the long-term behavior of the mortars. The porosity and water absorption results at 28 and 60 days are shown in Figure 7. The mortar incorporating WGP and MK exhibits a clear reduction in both porosity and water absorption with increasing curing time, indicating progressive densification of the cementitious matrix. In contrast, the control mortar shows only a marginal change between 28 and 60 days.

The improved performance of the blended mortar can be associated with ongoing pozzolanic reactions involving WGP and MK, which consume portlandite and promote the formation of additional hydration products, contributing to pore refinement and reduced pore connectivity. These observations are consistent with the trends identified in the paste-level analyses, including the reduced CH content observed by XRD and TGA/DSC and the more homogeneous microstructural features evidenced by SEM. Overall, the combined results indicate that the incorporation of WGP and MK enhances not only the mechanical performance but also the durability-related properties of the mortars through modifications of the cementitious matrix and its pore structure.

While specific durability tests—such as carbonation, chloride penetration, or sulfate attack—exceed the scope of this initial mechanical and microstructural characterization, the measured water absorption and porosity provide essential indirect indicators of the material’s long-term performance. The significantly lower values observed in the WGP–MK mortars demonstrate the achievement of a highly densified matrix with reduced interconnectivity, which is expected to enhance resistance against fluid ingress and aggressive ionic species. To further validate these findings, comprehensive durability campaigns including accelerated carbonation and chloride migration assays are currently being executed as part of the next phase of this research. These ongoing studies will provide a definitive assessment of the long-term performance and life-cycle sustainability of these optimized ternary systems [28].

## 4. Conclusions

The investigation into the individual and combined effects of WGP and MK on the mechanical and microstructural properties of cement-based systems leads to several key findings. The results demonstrate that while MK significantly enhances early-age compressive strength due to its high pozzolanic reactivity, WGP contributes more effectively at later stages through the gradual refinement of the pore structure. The ternary system containing 10 wt.% WGP and 5 wt.% MK (10WGP5MK) exhibited the best overall performance, achieving a compressive strength of 79.99 MPa at 28 days. This represents a substantial improvement over the control sample and the respective binary blends, identifying this specific dosage as the optimal balance for synergistic hydration.

The comprehensive analysis confirms a fundamental synergistic effect between WGP and MK derived from their complementary reaction kinetics. This interaction was quantitatively validated by TGA, which revealed a significant reduction in portlandite (CH) content of up to 52% in the ternary system. This decrease, normalized to the anhydrous cement weight, provides definitive evidence of the high pozzolanic efficiency of the blended binder. While the high aluminosilicate reactivity of MK compensates for the slower initial activity of the glass, the WGP ensures continuous microstructural densification through the gradual release of reactive silica. This synergy not only achieves higher mechanical strength but also promotes a more homogeneous distribution of hydration products, optimizing the material’s matrix compared to traditional binary systems.

Microstructural and structural analysis through SEM, XRD, and physical gas adsorption further confirmed that this synergy leads to the formation of additional C–S–H and C–A–S–H gels. Regarding the pore architecture, the nitrogen adsorption tests revealed a complex redistribution within the mesoporous range (2–50 nm) according to the IUPAC classification. Although the ternary blends showed intermediate total pore volumes, they achieved a more disconnected and refined pore network. This evolution of the internal structure reduces the connectivity of capillary pores, directly correlating with the observed reduction in water absorption and porosity over time.

Finally, the replacement of cement with WGP and MK offers a superior technical and sustainable alternative for the construction industry. The enhanced mechanical and durability-related indicators suggest a high potential for long-term performance. To complement these findings, further experimental campaigns focusing on accelerated carbonation and chloride migration are currently being executed as part of the next phase of this research. These ongoing studies will provide a comprehensive assessment of the long-term durability and life-cycle benefits of these optimized ternary cementitious systems, advancing circular economy goals through the efficient valorization of industrial by-products.

## Figures and Tables

**Table 1:** Chemical composition of precursor materials (wt.%).

Material	SiO_2_	Al_2_O_3_	Fe_2_O_3_	CaO	SO_3_	Na_2_O	K_2_O	MgO	TiO_2_	MnO_3_
PC	19.2	3.1	2.6	69.9	3.4	0.1	0.1	0.7	0.2	0.1
WGP	71.0	4.0	0.5	12.6	0.3	10.3	0.9	0.2	0.2	0.0
MK	54.8	26.2	1.3	8.0	1.6	1.9	0.2	0.5	0.4	2.0

**Table 2:** Particle size distribution parameters of cementitious materials (µm).

Material	d10	d50	d90
PC	2.0	10.1	29.2
WGP	2.4	10.0	25.0
MK	2.5	12.1	39.4

**Table 3:** Mixture proportions of pastes (%).

Mixture	PC	WGP	MK	w/cm
Control	100	0	0	0.32
15WGP	85	15	0	0.32
15MK	85	0	15	0.32
10WGP5MK	85	10	5	0.32
5WGP10MK	85	5	10	0.32

**Table 4:** Mass loss (%) in different temperature ranges from TGA analysis.

Paste	25–110 °C	110–400 °C	400–500 °C	600–750 °C
Control	3.6	6.8	3.1	12.4
15WGP	3.3	6.4	1.7	12.1
10WGP5MK	3.7	6.8	1.5	12.0

**Table 5:** Pore structure parameters of studied pastes at 28 days.

Mixture	Specific Surface Area (m^2^/g)	Total Pore Volume (cm^3^/g)	Average Pore Size (nm)
Control	18.42	0.0747	8.11
15WGP	12.39	0.0489	7.89
10WGP5MK	13.64	0.0599	8.74

## Data Availability

The original contributions presented in the study are included in the article. Further inquiries can be directed to the corresponding authors.

## References

[1] Andrew R.M. (2018). Global CO_2_ Emissions from Cement Production, 1928–2017. Earth Syst. Sci. Data.

[2] Liao S., Wang D., Xia C., Tang J. (2022). China’s Provincial Process CO_2_ Emissions from Cement Production during 1993–2019. Sci. Data.

[3] Cheng D., Reiner D.M., Yang F., Cui C., Meng J., Shan Y., Liu Y., Tao S., Guan D. (2023). Projecting Future Carbon Emissions from Cement Production in Developing Countries. Nat. Commun..

[4] Miller S.A., John V.M., Pacca S.A., Horvath A. (2018). Carbon Dioxide Reduction Potential in the Global Cement Industry by 2050. Cem. Concr. Res..

[5] Scrivener K.L., John V.M., Gartner E.M. (2018). Eco-Efficient Cements: Potential Economically Viable Solutions for a Low-CO_2_ Cement-Based Materials Industry. Cem. Concr. Res..

[6] Pamenter S., Myers R.J. (2021). Decarbonizing the Cementitious Materials Cycle: A Whole-Systems Review of Measures to Decarbonize the Cement Supply Chain in the UK and European Contexts. J. Ind. Ecol..

[7] Shah I.H., Miller S.A., Jiang D., Myers R.J. (2022). Cement Substitution with Secondary Materials Can Reduce Annual Global CO_2_ Emissions by up to 1.3 Gigatons. Nat. Commun..

[8] Oyejobi D.O., Firoozi A.A., Fernández D.B., Avudaiappan S. (2024). Integrating Circular Economy Principles into Concrete Technology: Enhancing Sustainability through Industrial Waste Utilization. Results Eng..

[9] Onsongo S.K., Olukuru J., Mwabonje O. (2025). Circular Economy in the Cement Industry: A Systematic Review of Sustainability Assessment and Justice Considerations in Local Community Development. Circ. Econ. Sustain..

[10] Li Q., Qiao H., Li A., Li G. (2022). Performance of Waste Glass Powder as a Pozzolanic Material in Blended Cement Mortar. Constr. Build. Mater..

[11] Muhedin D.A., Ibrahim R.K. (2023). Effect of Waste Glass Powder as Partial Replacement of Cement & Sand in Concrete. Case Stud. Constr. Mater..

[12] Dvořák K., Dolák D., Dobrovolný P. (2017). The Improvement of the Pozzolanic Properties of Recycled Glass during the Production of Blended Portland Cements. Procedia Eng..

[13] Du H., Tan K.H. (2014). Waste Glass Powder as Cement Replacement in Concrete. J. Adv. Concr. Technol..

[14] Borges A.L., Soares S.M., Freitas T.O.G., Junior A.O., Ferreira E.B., Ferreira F.G.S. (2021). Evaluation of the Pozzolanic Activity of Glass Powder in Three Maximum Grain Sizes. Mater. Res..

[15] Nahi S., Leklou N., Khelidj A., Oudjit M.N., Zenati A. (2020). Properties of Cement Pastes and Mortars Containing Recycled Green Glass Powder. Constr. Build. Mater..

[16] Hassani M.S., Matos J.C., Zhang Y., Teixeira E.R. (2023). Green Concrete with Glass Powder—A Literature Review. Sustainability.

[17] Khaled Z., Mohsen A., Soltan A.M., Kohail M. (2023). Optimization of Kaolin into Metakaolin: Calcination Conditions, Mix Design and Curing Temperature to Develop Alkali Activated Binder. Ain Shams Eng. J..

[18] Geu M.J., Zhuge Y., Ma X., Pham T.M. (2025). Optimising Calcination Temperature for High Reactivity Metakaolin: Influence on Amorphous Content, Mineralogy and Microstructure. Constr. Build. Mater..

[19] Li W., Hua L., Shi Y., Wang P., Liu Z., Cui D., Sun X. (2022). Influence of Metakaolin on the Hydration and Microstructure Evolution of Cement Paste during the Early Stage. Appl. Clay Sci..

[20] Li C., Fan Y., Yu J. (2020). Compressive Strength and Pore Structure of Cement Paste with Metakaolin. IOP Conf. Ser. Earth Environ. Sci..

[21] Skibsted J., Snellings R. (2019). Reactivity of Supplementary Cementitious Materials (SCMs) in Cement Blends. Cem. Concr. Res..

[22] Jahami A., Frangieh H., Assaad J., Alkhatib A., Avci-Karatas C., Chieffo N. (2025). Experimental and Numerical Study Assessing the Synergistic Effect of Metakaolin and Waste Glass on the Concrete Mechanical and Structural Properties. Buildings.

[23] Nassar R.U.D., Saeed D., Ghebrab T., Room S., Deifalla A., Al Amara K. (2024). Heat of Hydration, Water Sorption and Microstructural Characteristics of Paste and Mortar Mixtures Produced with Powder Waste Glass. Cogent Eng..

[24] Chandra Sekhar M., Kumar M.H., Lova Raju S., Saikrishnamacharyulu I. (2023). Influence of Metakaolin and Glass Powder on Mechanical Behaviour of Concrete. Mater. Today Proc..

[25] Akinpelu M.A., Amao A.O., Salman A.S.M., Gabriel D.S. (2025). Sustainable Self-Compacting Concrete: A Study on the Combined Effects of Waste Glass Powder and Metakaolin as Cement Replacements. Res. Eng. Struct. Mater..

[26] Scrivener K., Snellings R., Lothenbach B. (2016). A Practical Guide to Microstructural Analysis of Cementitious Materials.

[27] Leal R.B., Morais C.R.d.S., Santos K.D.S. (2024). Assessing the Impact of Waste Glass and Metakaolin on the Durability and Mechanical Strength Properties of Concrete. Matéria.

[28] Wang Y., Zhu L., Xue Y. (2026). A Comprehensive Review on Dual-Pathway Utilization of Coal Gangue Concrete: Aggregate Substitution, Cementitious Activity Activation, and Performance Optimization. Buildings.

[29] Barbhuiya S., Kanavaris F., Ashish D.K., Tu W., Das B.B., Adak D. (2025). Ground Waste Glass as a Supplementary Cementitious Material for Concrete: Sustainable Utilization, Material Performance and Environmental Considerations. J. Sustain. Cem. Based. Mater..

[30] (2021). Specification for Standard Sand.

[31] Michel L., Sanner A., Zunino F., Flatt R.J., Kammer D.S. (2026). Contact Point Geometry Governs Structural Buildup at Rest in Portland Cement–Limestone Blends. J. Am. Ceram. Soc..

[32] Kovářík T., Bělský P., Novotný P., Říha J., Savková J., Medlín R., Rieger D., Holba P. (2015). Structural and Physical Changes of Re-Calcined Metakaolin Regarding Its Reactivity. Constr. Build. Mater..

[33] (2020). Practice for Mechanical Mixing of Hydraulic Cement Pastes and Mortars of Plastic Consistency.

[34] (2020). Test Method for Compressive Strength of Hydraulic Cement Mortars (Using 2-in. or [50-Mm] Cube Specimens).

[35] (2020). Standard Test Method for Flow of Hydraulic Cement Mortar.

[36] (2021). Test Method for Flexural Strength of Hydraulic-Cement Mortars.

[37] (2024). Test Method for Compressive Strength of Hydraulic-Cement Mortars (Using Portions of Prisms Broken in Flexure).

[38] (2021). Test Method for Density, Absorption, and Voids in Hardened Concrete.

[39] Krajči Ľ., Mojumdar S.C., Janotka I., Puertas F., Palacios M., Kuliffayová M. (2015). Performance of Composites with Metakaolin-Blended Cements. J. Therm. Anal. Calorim..

[40] Mehta P.K., Monteiro P.J.M. (2006). Concrete: Microstructure, Properties, and Materials.

[41] Gu Y., Zhang P., Lv R., Sun Q., Sun R., Xu S. (2025). The Effects of Two Typical Waste Glass Powders on the Hydration Properties, Kinetic Characteristics and Microstructure of Blended Cement. Ceram. Int..

[42] El-Diadamony H., Amer A.A., Sokkary T.M., El-Hoseny S. (2018). Hydration and Characteristics of Metakaolin Pozzolanic Cement Pastes. HBRC J..

[43] Zajac M., Skibsted J., Durdzinski P., Bullerjahn F., Skocek J., Ben Haha M. (2020). Kinetics of Enforced Carbonation of Cement Paste. Cem. Concr. Res..

[44] da Silva Andrade D., da Silva Rêgo J.H., Morais P.C., de Mendonça Lopes A.N., Rojas M.F. (2019). Investigation of C-S-H in Ternary Cement Pastes Containing Nanosilica and Highly-Reactive Supplementary Cementitious Materials (SCMs): Microstructure and Strength. Constr. Build. Mater..

[45] Thommes M., Kaneko K., Neimark A.V., Olivier J.P., Rodriguez-Reinoso F., Rouquerol J., Sing K.S.W. (2015). Physisorption of Gases, with Special Reference to the Evaluation of Surface Area and Pore Size Distribution (IUPAC Technical Report). Pure Appl. Chem..

[46] Wang L., Xue Y., Zhu L., Cao X., Li X., Ranjith P.G. (2026). Fracture Evolution of Granite under Cyclic Thermal Shocks: Effects of Liquid Nitrogen Cooling on Strength, Toughness, and Acoustic Emission Characteristics. Therm. Sci. Eng. Prog..

[47] Ortega J., Letelier V., Solas C., Miró M., Moriconi G., Climent M., Sánchez I. (2018). Influence of Waste Glass Powder Addition on the Pore Structure and Service Properties of Cement Mortars. Sustainability.

[48] Islam G.M.S., Rahman M.H., Kazi N. (2017). Waste Glass Powder as Partial Replacement of Cement for Sustainable Concrete Practice. Int. J. Sustain. Built Environ..

[49] Kocak Y. (2020). Effects of Metakaolin on the Hydration Development of Portland–Composite Cement. J. Build. Eng..

[50] Dobiszewska M., Pichór W., Tracz T., Petrella A., Notarnicola M. (2023). Effect of Glass Powder on the Cement Hydration, Microstructure and Mechanical Properties of Mortar. Mater. Proc..

